# What fMRI studies say about the nature of the psychedelic effect: a scoping review

**DOI:** 10.3389/fnins.2025.1606798

**Published:** 2025-07-01

**Authors:** Michal Beneš, Tomáš Páleníček, Jiří Horáček

**Affiliations:** ^1^Department of Psychiatry and Medical Psychology, Third Faculty of Medicine, Charles University, Prague, Czechia; ^2^Center for Advanced Studies of Brain and Consciousness, National Institute of Mental Health, Klecany, Czechia; ^3^Psychedelic Research Center, National Institute of Mental Health, Klecany, Czechia

**Keywords:** psychedelics, psilocybin, LSD, ayahuasca, DMT, fMRI, functional connectivity, entropy

## Abstract

Research on psychedelic drugs, such as psilocybin, LSD or DMT, is a burgeoning field, with an increasing number of studies showing their promise in treatment of mental disorders as well as examining their mechanism of action. Determining their effect on the brain is crucial from clinical standpoint, but also offers highly promising avenues of advancement in basic neuroscience—functional magnetic resonance imaging (fMRI) is one of the most useful techniques to do so, with a number of newly published studies increasing every year. Here we present a scoping review of existing fMRI studies of serotonergic psychedelics to date, with a focus on finding unifying themes among them, in order to comprehensively grasp current directions within this field. We cluster the existing studies by fMRI modality and find several lines of developing concepts complementing the established models of psychedelic actions on the brain: namely, we describe a general picture of de-differentiation with the default mode network at its core captured by a diverse array of different techniques, complex changes to the thalamus, amygdala and medial temporal lobe structures, and the importance of the phenomenon of ego dissolution. Finally, contrasts to phenomenologically similar states and the successful process of anchoring fMRI findings to other markers are discussed.

## 1 Introduction

The research into psychedelics is undergoing a resurgence. Accumulating clinical data point to a great potential of these substances in treatment of a range of psychiatric disorders. If proven effective, psychedelic drugs would provide a sorely needed addition to the armamentarium of psychiatric therapies, which currently still remain inadequate for an unacceptably large portion of patients—as an example, in a respected study for treatment of depression, a full third of patients did not achieve remission (Gaynes et al., 2009). Psychedelics with their radically different mechanism of action thus present an opportunity to narrow or even fill this gap. Not just in treatment-resistant depression—research shows promise in addictions, OCD, existential distress in terminal conditions (Nichols, 2016), anorexia nervosa (Peck et al., 2023), depressive episodes in type II bipolar disorder (Aaronson et al., 2024), and even beyond psychiatry as in migraine (Schindler et al., 2021), cluster headache (Madsen et al., 2024) and more.

With such promising empirical data, research into elucidating mechanisms of action becomes indispensable. It has long been known that the key receptor target for classical serotonergic psychedelics is the 5HT2A receptor (Nichols, 2016), but that still tells us very little about the complex effect these compounds clearly have on the human brain on all levels, from deeper molecular mechanisms within cells to neuron-to-neuron interaction, regional activity changes, whole-brain function changes all the way to subjective effects and observable behavior. Fortunately, substantial progress is being made on all of these levels. Especially when looking at their whole-brain and regional effects, it can be appreciated how psychedelics can also serve as a tool for informing work in more theoretical areas of neuroscience such as consciousness research (Rankaduwa and Owen, 2023), painting a rich picture of the human brain function all the way from health to disease and how that function can be externally perturbed and further studied that way.

Functional magnetic resonance imaging (fMRI) allows for investigating multiple aspects of brain function on this large-scale level, depending on the modality used. Task-based fMRI studies visualize activations of brain areas in response to a defined stimulus (e.g., presentation of a picture). On the other hand, resting-state fMRI studies investigate functional connectivity of brain regions without any outside stimulus, thus reflecting (as the name suggests) communication between brain areas at rest. This way, organization of the brain into so called canonical resting state networks emerges. Functional connectivity can be further expanded upon in studies of effective connectivity—this method employs further statistical methods to determine the direction and valence (excitatory or inhibitory) of the signal (Chen and Glover, 2015). A different approach is presented by a heterogenous group of methods we collectively call here global measures, which instead of subdividing the brain into areas study its functional integration as a whole.

There have been groundbreaking psychedelic fMRI studies made in the previous decades, making findings that form the basis of current research and helping formulate the general contemporary theories of psychedelic action on the brain—the most frequently discussed being the Cortico-Striatal-Thalamo-Cortical (CSTC) model and the Relaxed Beliefs Under Psychedelics (REBUS) model, as well as the more recent Cortico-Claustro-Cortical (CCC) model and a number of others. More specifically, the CSTC model posits psychedelics to disinhibit the thalamus via direct 5HT2A receptor action, causing more excitatory action on the cortex, and thus weakening its function as a filter and allowing more information to pass through—e.g. as visual hallucinations (Vollenweider and Geyer, 2001). The REBUS model builds on the predictive coding principle and proposes that psychedelic drugs cause decrease in confidence in prior beliefs though increase in brain entropy (see section 3.1 for definition and discussion of the term), and thus allow for their revision (Carhart-Harris and Friston, 2019). Other models have been proposed as well, such as the CCC model, which attributes the observed desynchronization of canonical resting state networks (such as the DMN) to their functional decoupling from the claustrum, which is rich in the 5HT2A receptors (Doss et al., 2022). For a review of these models of psychedelic action see Doss et al. (2022).

As of now, as the field is further maturing, a sufficient number of papers exists to allow for formulation of broader conceptual “flows” within the current discourse independently of the theoretical models, offering a broad snapshot of the current state of knowledge. There has already been a number of review and systematic review articles conducted in this field, bringing into focus various aspects of the developing field: a review (McCulloch et al., 2022b) and a systematic review (Linguiti et al., 2023) of psychedelic neuroimaging mainly focused on methodology, a systematic review specifically on the DMN modulation (Gattuso et al., 2022), a meta-analysis of functional imaging (Castelhano et al., 2021), a review of neuroimaging in psilocybin-based psychotherapy (Gill et al., 2022), and more (Müller and Borgwardt, 2019; Soares et al., 2023). Also see Vollenweider and Preller (2020) and Kwan et al. (2022) for broader reviews of psychedelic mechanisms of action. However, to the best of our knowledge, a work solely focused at summarizing all of the current themes within the findings in psychedelic fMRI has been missing. To remediate this gap, we have conducted the present scoping review, which we believe would be valuable especially for new researchers seeking to enter the field. We would also like to bring attention to a very recent review published that brings valuable perspective on differences between fMRI (and PET) findings between individual psychedelic substances (Frautschi et al., 2024), whereas we choose to treat the serotonergic psychedelics as a whole.

For the sake of intelligibility, we cluster the results to several categories. There are multiple possible ways to do this: by specific substances, by acute vs. longitudinal effects, by population and more. We chose a rough clustering by different fMRI techniques as outlined above—each of them examines different aspects of brain function not available to the others, thus reflecting distinct processes (the line between these clusters naturally becoming blurry in multiple instances). In addition, we add a specific section dedicated to ego dissolution, which seems to be emerging as a key subjective phenomenon within the psychedelic experience, and which on its own carries enough fMRI correlates to be discussed here separately. The findings we discuss in the narrative text are those that have been detected by multiple studies, or are otherwise in line with other research.

## 2 Methods

**Figure 1:**
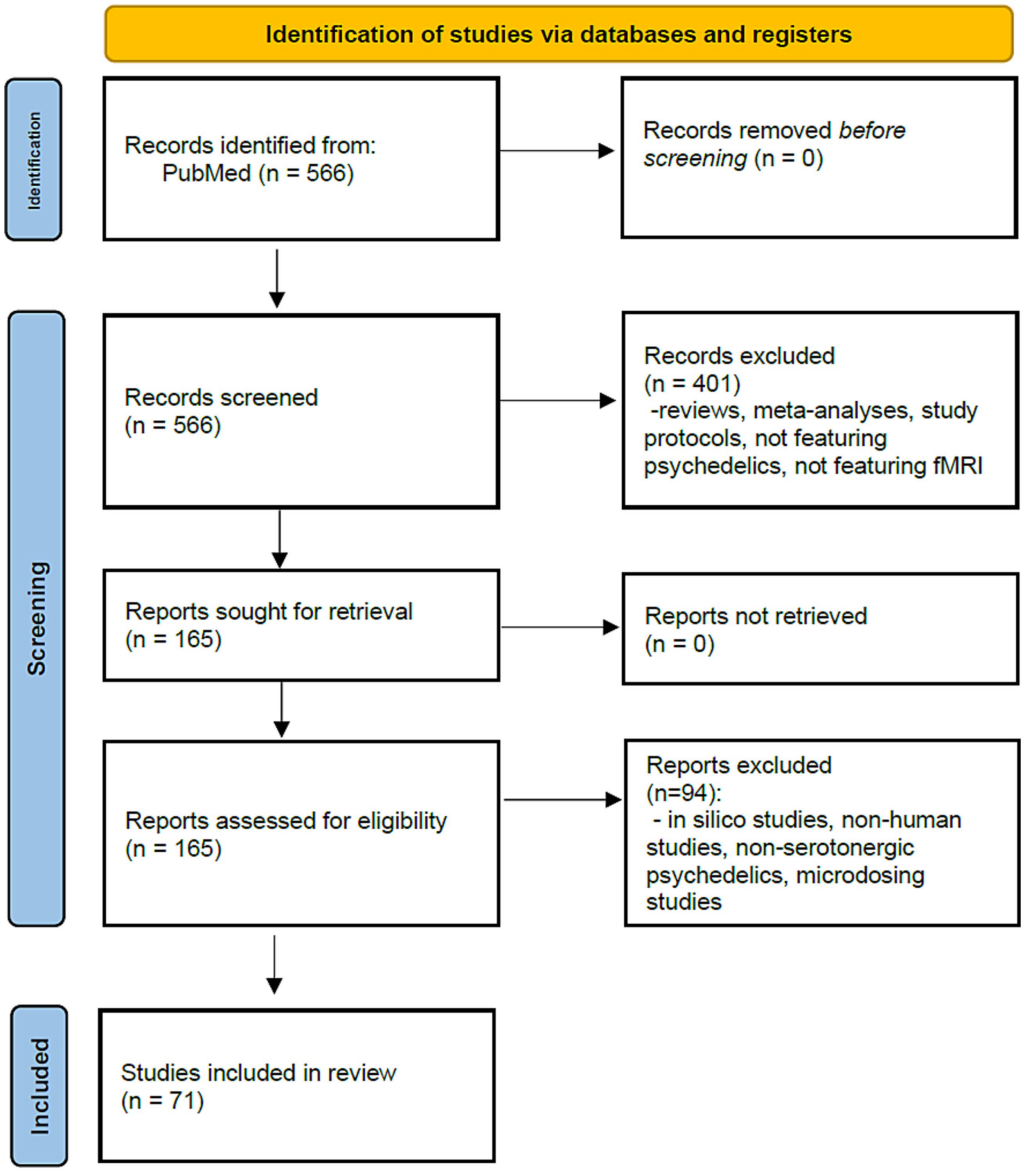
PRISMA flow diagram for study selection and exclusion process.

To conduct this scoping review, we carried out search of the existing literature on fMRI imaging of the effect of serotonergic psychedelics, published as of time of search. Inclusion criteria were: articles in English language, use of fMRI, direct analysis of fMRI data from human subjects who received psilocybin, LSD, DMT, ayahuasca or mescaline. The literature available on Pubmed was searched in September 2024. The following search string was applied: *(psychedelic OR psilocybin OR lsd OR mescaline OR ayahuasca OR dimethyltryptamine) AND (fmri OR “functional magnetic” OR BOLD OR “arterial spin labeling”)* In total, 566 references were obtained. See Figure 1 for PRISMA flow diagram of study selection and for exclusion criteria (Page et al., 2021). Articles selected for analysis were extracted and organized using a reference manager, read in full text and reviewed by M. B. and J. H. with disagreements resolved by discussion, and with all authors then contributing to the information synthesis.

## 3 Results

A total of 71 original studies were reviewed in full text. Thirty five of these studies examined psilocybin, 30 LSD, 8 ayahuasca and 5 DMT. There was no fMRI study concerning mescaline. As for clustering by fMRI techniques we use in this review, 27 studies examined functional connectivity, 22 global measures, 15 were task-based, and 7 examined effective connectivity. Multiple studies examined more than one substance, and some studies belong into more than one fMRI modality. Overview of findings from all studies reviewed is presented in Table 1. As mentioned above, not all of these studies are discussed in the narrative text, as our main focus was to capture broader concepts. Vast majority of studies examined the acute effect of the substances, i.e., fMRI scans being acquired during intoxication; for studies where this is not the case (e.g., scans made before and after intoxication), this fact is highlighted in Table 1. Methodological parameters of all studies reviewed are presented in Table 2.

**Table 1:** Summary of findings of and techniques utilized in the reviewed studies.

Authors	Substance	Method details	Main finding
Global measures			
Tagliazucchi et al. (2014)	Psilocybin 2 mg i.v.	Dynamic rsFC	Increased time variability and entropy in hippocampi and ACC; increased number of connectivity motifs
Lord et al. (2019)	Psilocybin 2 mg i.v.	Leading Eigenvector Dynamics Analysis (LEiDA)	Destabilization of frontoparietal subsystem and instead increase in global coherence
Preller et al. (2020)	Psilocybin 14 mg/70 kg p.o.	Global brain connectivity	Increased sensory global connectivity and decreased associative global connectivity; baseline connectivity associated with magnitude of changes
Olsen et al. (2022)	Psilocybin 0.24 ± 0.04 mg/kg	Leading Eigenvector Dynamics Analysis (LEiDA)	Negative correlation of frontoparietal state occurrence with psilocin plasma levels and subjective drug effects, positive correlation of global brain state with psilocin plasma levels and subjective drug effects
Daws et al. (2022)	Psilocybin 10 mg and 25 mg p.o. (open label); Psilocybin 2× 25 mg p.o. (DB-RCT)	Brain network modularity; measured after intoxication	Brain network modularity was decreased 1 day after treatment, which correlated with alleviation of depressive symptoms after 6 months
Mortaheb et al. (2024)	Psilocybin 0.17 mg/kg	Time-varying functional connectivity analysis	Increased averaged brain functional connectivity, hyperconnected pattern with low BOLD signal amplitude, correlation with feelings of oceanic boundlessness and visionary restructuralization
Varley et al. (2020)	LSD 75 μg i.v., psilocybin 2 mg i.v.	Fractal dimension of functional connectivity	Increased fractal dimension of networks and BOLD signal, suggestive of increased entropy, localized to dorsal attention network
Girn et al. (2022)	LSD 75 μg i.v., psilocybin 2 mg i.v.	Gradient-mapping analysis	Flattening of principal gradient of cortical connectivity
Singleton et al. (2022)	LSD 75 μg i.v., psilocybin 2 mg i.v.	Network control analysis	Reduction of control energy needed for transitioning between brain states
Luppi et al. (2023)	LSD 75 μg i.v., psilocybin 2 mg i.v., DMT 20 mg i.v., ayahuasca (2.2 mL/kg containing 0.8 mg/mL of DMT and 0.21 mg/mL of harmine) (10 substances in total)	Effects of different substances and cortical distributions of neurotransmitters on FC	Effects of compared substances organized along the principal cortical gradient
Tagliazucchi et al. (2016)	LSD 75 μg i.v.	Global functional connectivity	Increase in global integration by increase in connectivity between normally distinct networks, correlating with ego dissolution
Lebedev et al. (2016)	LSD 75 μg i.v.	Sample entropy	Increased entropy, which predicted increased openness after 2 weeks
Atasoy et al. (2017)	LSD 75 μg i.v.	Connectome-harmonic decomposition	Expansion of the repertoire of active brain states, move toward criticality
Preller et al. (2018b)	LSD 100 μg p.o.	Global brain connectivity	Increased sensory-somatomotor whole-brain and thalamic connectivity, decreased associative connectivity
Luppi et al. (2021)	LSD 75 μg i.v.	Dynamic rsFC	Weakening of the relationship between functional and anatomic connectivity; ego-dissolution predicted by increase in small-world organization; also decrease of FC of mPFC
Lawn et al. (2022)	LSD 75 μg i.v.	Receptor-enriched analysis of functional connectivity by targets	Serotonergic system is associated with perception changes, dopaminergic system with perceived selfhood and cognition
Viol et al. (2017)	Ayahuasca (2.2 mL/kg containing 0.8 mg/mL of DMT and 0.21 mg/mL of harmine)	Shannon entropy	Increase in entropy and local network integration, decrease in global integration
Mallaroni et al. (2024)	Ayahuasca (mean 24 mL, containing 0.14 mg/mL of DMT, 4.50 mg/mL of harmine, 0.51 mg/mL of harmaline, and 2.10 mg/mL of tetrahydroharmine)	Static and dynamic functional connectivity	Interindividual differences in higher functional connectivity motifs predict perceptual effects
Timmermann et al. (2023)	DMT 20 mg i.v.	Global functional connectivity	Increase in global functional connectivity, disintegration and desegregation of networks, compression of principal cortical gradient
Singleton et al. (2023)	DMT 20 mg i.v.	Network control analysis	Reduction of control energy needed for transitioning between brain states
Pasquini et al. (2024)	DMT 20 mg i.v.	Dynamic activity matrix	Increased superior temporal lobe activity; Decreased hippocampal and medial parietal cortex activity, which correlated with meaningfulness of the experience
Vohryzek et al. (2024)	DMT 7/14/18/20 mg i.v.	Connectome-harmonic decomposition	Move toward higher frequencies, divergence from structural connectome, move toward criticality
Resting-state functional connectivity			
Carhart-Harris et al. (2012a)	Psilocybin 2 mg i.v.		Decreased CBF and BOLD especially in thalamus, ACC and PCC; decreased connectivity between mPFC and PCC
Carhart-Harris et al. (2013)	Psilocybin 2 mg i.v.		Decrease in anticorrelation of the DMN and task-positive network (increase in FC between them)
Roseman et al. (2014)	Psilocybin 2 mg i.v. (MDMA)		Increased between-network connectivity
Lebedev et al. (2015)	Psilocybin 2 mg i.v.		Measures of ego-dissolution correlated with decreased FC between MTL and the cortex, with disintegration of the salience network and with decreased interhemispheric communication
Carhart-Harris et al. (2017)	Psilocybin 10 mg and 25 mg p.o.	Measured after intoxication	Increase in FC within DMN post-treatment in treatment-resistant depression
Smigielski et al. (2019)	Psilocybin 315 μg/kg p.o.		Decreased FC between mPFC and PCC, associated with ego-dissolution, which predicted positive changes in psychosocial functioning after 4 months
Barrett et al. (2020a)	Psilocybin 25 mg/70 kg p.o.	Measured after intoxication	Increase in significant connections after 1 week and 1 month
Barrett et al. (2020b)	Psilocybin 10 mg/70 kg p.o.		Decreased BOLD variance and LFF amplitude in the claustra, predicted by subjective effects; various changes in their network connectivity
Madsen et al. (2021)	Psilocybin 0.2–0.3 mg/kg p.o.		Psilocin plasma levels and subjective effects negatively correlate with network integrity, positively with CEN and DAN desegregation and connectivity to rest of the brain
Gaddis et al. (2022)	Psilocybin 10 mg/70 kg p.o.		Decreased FC between mediodorsal nucleus of the thalamus and the pulvinar with the DMN and visual network; magnitude of these changes correlated with subjective effects
McCulloch et al. (2022a)	Psilocybin 0.2–0.3 mg/kg p.o.	Measured after intoxication	Decrease of CEN FC after 1 week, this predicted increase in mindfulness after 3 months
Madsen et al. (2024)	Psilocybin 0.14 mg/kg 3 times	Measured after intoxication	Negative correlation of hypothalamic-diencephalic FC and percent change in chronic cluster headache attack frequency
Siegel et al. (2024)	Psilocybin 25 mg p.o. (Methylphenidate)	Longitudinal rsFC tracking; measured also after intoxication	Reduced correlations within and anticorrelations between networks, most pronounced in the DMN; decrease in FC between DMN and the hippocampus lasting weeks
Moujaes et al. (2024)	LSD 100 μg p.o., psilocybin 0.2 mg/kg p.o.		Psilocybin and LSD effect determined by machine learning differed from hypnosis and meditation
Carhart-Harris et al. (2016)	LSD 75 μg i.v.		Decreased FC between PHC and retrosplenial cortex correlated with ratings of ego-dissolution and altered meaning
Tagliazucchi et al. (2016)	LSD 75 μg i.v.		Increased FC between association cortex and the thalamus; increase in global FC correlating with measures of ego-dissolution
Speth et al. (2016)	LSD 75 μg i.v.		Decreased FC within the DMN, which correlated with decrease of language references to the past
Müller et al. (2017)	LSD 100 μg p.o.		Increased FC between thalamus and cortical areas; the increase in FC specifically with fusiform gyrus and insula correlated with subjective auditory and visual effects
Müller et al. (2018)	LSD 100 μg p.o.		Decrease of FC within visual, sensomotor, auditory and default-mode networks, and increase in FC within all networks and between networks and the thalamus, striatum, precuneus and ACC
Avram et al. (2022)	LSD 100 μg p.o. (MDMA, D-amphetamine)		Increased FC between thalamus and auditory, sensorimotor (all substances) and SN (LSD only)
Dai et al. (2023)	LSD 75 μg i.v. (NO, ketamine)		All 3 substances decreased within-network connectivity and increased between-network connectivity
Pizzi et al. (2023a)	LSD 75 μg i.v.		Increase in FC and signal amplitude in default-mode and attention networks rich with 2A receptors; decrease of FC and signal amplitude in limbic areas rich in 1A receptors
Pizzi et al. (2023b)	LSD 75 μg i.v.		Increased FC between thalamamic ventral nuclei, pulvinar and sensory and association cortex
Shukuroglou et al. (2023)	Psilocybin 10 mg and 25 mg p.o.	Measured after intoxication	Decrease of FC between nucleus accumbens and the DMN after music listening during dosing for treatment-resistant depression, compared to dosing without music listening
Palhano-Fontes et al. (2015)	Ayahuasca (2.2 mL/kg containing 0.8 mg/mL of DMT and 0.21 mg/mL of harmine)		Decrease of activity in the DMN and decrease of FC between PCC and precuneus
Sampedro et al. (2017)	Ayahuasca (148 ± 29 mL, containing 0.3 mg/mL DMT, 0.86 mg/mL harmine, 0.17 mg/mL tetrahydroharmine, and 0.04 mg/mL harmaline)	Measured after intoxication	Postacute increase of ACC FC of PCC and right MTL; changes predicted with increase in nonjudging after 2 months
Pasquini et al. (2020)	Ayahuasca (1 mL/kg, containing 0.36 mg/mL of N, N-DMT, 1.86 mg/mL of harmine, 0.24 mg/mL of harmaline, and 1.20 mg/mL of tetrahydroharmine)	Measured after intoxication	Increase in FC of ACC within the salience network and decrease of FC of PCC within the DMN
Effective connectivity			
Kraehenmann et al. (2015a)	Psilocybin 0.16 mg/kg p.o.		Decrease by threat-induced connectivity from amygdala to V1 visual cortex
Stoliker et al. (2024)	Psilocybin 0.2 mg/kg p.o.		Decrease of EC from cortical regions to the amygdala, with the exception of the DMN
Kaelen et al. (2016)	LSD 75 μg i.v.		Increase of FC and EC from PHC to visual cortex during music listening
Preller et al. (2019)	LSD 100 μg p.o.		Increase of EC from thalamus to PCC and decrease of EC from striatum ventrale to the thalamus
Stoliker et al. (2023)	LSD 100 μg p.o.		Change of inhibitory EC from SN to DMN to excitatory and decrease of inhibitory EC from DMN to DAN during ego dissolution
Avram et al. (2023)	LSD 100 μg p.o. (MDMA, D-amphetamine)		Increase of EC from thalamus to unimodal cortex by all substances, LSD additionally increased EC from thalamus to transmodal cortex
Bedford et al. (2023)	LSD 100 μg p.o.	Regression DCM	Stronger FC and EC with the exception of connections involving occipital and subcortical regions; perturbed excitation/inhibition balance
Task-based fMRI—activation studies			
Carhart-Harris et al. (2012b)	Psilocybin 2 mg i.v.		Activation in visual and other sensory areas while watching 15 positive autobiografical stimuli
Kraehenmann et al. (2015b)	Psilocybin 0.16 mg/kg p.o.		Decreased amygdala reactivity to negative and neutral stimuli
Preller et al. (2016)	Psilocybin 0.215 mg/kg p.o.		Decreased response to social exclusion in dorsal ACC and the middle frontal gyrus
Roseman et al. (2018)	Psilocybin 10 mg and 25 mg p.o.	Measured after intoxication	Increase of right amygdala reactivity to fearful and happy faces in treatment-resistant depression
Barrett et al. (2020a)	Psilocybin 25 mg/70 kg p.o.	Measured after intoxication	Decreased amygdala activation in response to faces and increased DMPFC and mOFC in response to emotionally conflicting stimuli 1 week after dosing
Mertens et al. (2020)	Psilocybin 10 mg and 25 mg p.o.	Measured after intoxication	Decreased connectivity of vmPFC with right amygdala during processing of fearful and neutral faces in treatment-resistant depression
Duerler et al. (2021)	Psilocybin 0.2 mg/kg p.o.		Decrease in activity in frontal areas, visual cortex and cerebellum in response to tactile mismatch responses
Pagni et al. (2024)	Psilocybin 25 mg/70 kg p.o.	Measured after intoxication	Increase of PFC and caudate and decrease in insula, motor cortex and cerebellum activity with visual and emotionally valenced stimuli in patients with alcohol use disorder
Mueller et al. (2017)	LSD 100 μg p.o.		Reduced amygdala and right mPFC reactivity to fearful faces
Preller et al. (2017)	LSD 100 μg p.o.		Induction of attribution of personal relevance to previously meaningless stimuli and modulated the processing of meaningful stimuli in dorsal ACC, PCC
Preller et al. (2018a)	LSD 100 μg p.o.		Reduction of activity in PCC and angular gyrus in social interaction task
Schmidt et al. (2018)	LSD 100 μg p.o.		In Go/No-Go Task, impaired inhibitory performance related to parahippocampal and left superior frontal gyrus activation
Duerler et al. (2020)	LSD 100 μg p.o.		Increased mPFC activity in social adaptation task
de Araujo et al. (2011)	Ayahuasca (2.2 mL/kg containing 0.8 mg/mL of DMT and 0.21 mg/mL of harmine)		Activation in V1 visual cortex during closed eyes imagery task to same degree as with eyes open (lease activations in occipital, temporal and frontal areas)
Arruda Sanchez et al. (2024)	Ayahuasca (30 mL/70 kg, corresponding to 0.333 mg/kg DMT, 0.473 mg/kg Harmine, 0.023 mg/kg Harmaline, and 0.470 mg/kg THH)		Decreased amygdala activation with implicit aversive stimulation in face recognition task

ACC, anterior cingulate cortex; BOLD, blood-oxygen-level-dependent imaging; CBF, cerebral blood flow; CEN, central executive network; DAN, dorsal attention network; DMN, default mode network; dmPFC, dorsomedial rpefrontal cortex; EC, effective connectivity; FC, functional connectivity; LFF, low frequency fluctuations; mOFC, medial orbitofrontal cortex; mPFC, medial prefrontal cortex; MTL, medial temporal lobe; PCC, posterior cingulate cortex; PFC, prefrontal cortex; PHC, parahippocampal cortex; SN, salience network; vmPFC, ventromedial prefrontal cortex.

**Table 2:** Summary of methodologies of reviewed studies.

Authors	Substance	Comparator condition, study design	Sample details	Timing of scan	Scanner information
Global Measures					
Tagliazucchi et al. (2014)	Psilocybin 2 mg i.v.	Placebo, within-subjects	15 healthy subjects, 13 males and 2 females, mean age = 32y	60 s infusion exactly 6 min after the start of the 12 min scan	3 T GE HDx system
Lord et al. (2019)	Psilocybin 2 mg i.v.	Placebo, within-subjects	15 healthy subjects, 13 males and 2 females, mean age = 32y	60 s infusion exactly 6 min after the start of the 12 min scan	3 T GE HDx system
Preller et al. (2020)	Psilocybin 14 mg/70 kg p.o.	Placebo, double-blinded, randomized, crossover design	23 healthy subjects, 12 males, 11 females, mean age = 26.3y	20, 40, and 70 min after treatment administration	3 T Philips Achieva
Olsen et al. (2022)	Psilocybin 0.24 ± 0.04 mg/kg	Ketanserin, crossover design	15 healthy subjects, 6 females, mean age 34.3y ± 9.8 years	Once before and approx. 40, 80, 130, and 300 min after administration	3 T Siemens Prisma scanner
Daws et al. (2022)	Psilocybin 10 mg and 25 mg p.o. (open label); Psilocybin 2× 25 mg p.o. (DB-RCT)	Baseline, open label trial and double-blind randomized controlled trial	16 subjects with TRD, 4 female, mean age = 42.75y, SD = 10.15 (open label); 22 subjects with TRD, 8 female, mean age = 44.5y, SD = 11.0 (DB-RCT)	At baseline and 1 day after second dosing (open label); At baseline and 3 weeks after second dosing (DB-RCT)	3 T Siemens Tim Trio
Mortaheb et al. (2024)	Psilocybin 0.17 mg/kg	Placebo, randomized	49 healthy subjects (psilocybin n = 22, 12 male; mean age = 23y, SD = 2.9; placebo n = 27, 15 male, age = 23.1y, SD = 3.8)	Subjects placed in scanner 40 min after administration, scans performed throughout a 1-h time window	7 T Siemens MAGNETOM scanner
Varley et al. (2020)	LSD 75 μg i.v., psilocybin 2 mg i.v.	Placebo, within-subjects (both substances)	20 healthy subjects, 4 females, mean age = 30.9y ± 7.8 (LSD), 15 healthy subjects, 13 males and 2 females, mean age = 32y (psilocybin)	60 min after infusion (LSD), 60 s infusion exactly 6 min after the start of the 12 min scan (psilocybin)	3 T GE HDx system
Girn et al. (2022)	LSD 75 μg i.v., psilocybin 2 mg i.v.	Placebo, within-subjects (both substances)	20 healthy subjects, 4 females, mean age = 30.9y ± 7.8 (LSD), 15 healthy subjects, 13 males and 2 females, mean age = 32y (psilocybin)	60 min after infusion (LSD), 60 s infusion exactly 6 min after the start of the 12 min scan (psilocybin)	3 T GE HDx system
Singleton et al. (2022)	LSD 75 μg i.v., psilocybin 2 mg i.v.	Placebo, within-subjects (both substances)	20 healthy subjects, 4 females, mean age = 30.9y ± 7.8 (LSD), 15 healthy subjects, 13 males and 2 females, mean age = 32y (psilocybin)	60 min after infusion (LSD), 60 s infusion exactly 6 min after the start of the 12 min scan (psilocybin)	3 T GE HDx system
Luppi et al. (2023)	LSD 75 μg i.v., psilocybin 2 mg i.v., DMT 20 mg i.v., ayahuasca (2.2 mL/kg containing 0.8 mg/mL of DMT and 0.21 mg/mL of harmine) (10 substances in total)	Placebo, within-subjects (LSD, psilocybin); Placebo, single-blind, counter-balanced (DMT); Baseline, open label (ayahuasca)	(Carhart-Harris et al., 2016 for LSD; Carhart-Harris et al. (2012a) for psilocybin; Timmermann et al., 2023 for DMT; Viol et al., 2017 for ayahuasca)		
Tagliazucchi et al. (2016)	LSD 75 μg i.v.	Placebo, within-subjects	20 healthy subjects, 4 females, mean age = 30.9y ± 7.8	60 min after infusion	3 T GE HDx system
Lebedev et al. (2016)	LSD 75 μg i.v.	Placebo, within-subjects	20 healthy subjects, 4 females, mean age = 30.9y ± 7.8	60 min after infusion	3 T GE HDx system
Atasoy et al. (2017)	LSD 75 μg i.v.	Placebo, within-subjects	20 healthy subjects, 4 females, mean age = 30.9y ± 7.8 (12 used)	60 min after infusion	3 T GE HDx system
Preller et al. (2018b)	LSD 100 μg p.o.	Placebo/ketanserin, double-blind, randomized, crossover design	24 healthy subjects, 5 females, mean age = 25.00y; SD = 3.60	75 and 300 min after treatment administration	3 T Philips Achieva
Luppi et al. (2021)	LSD 75 μg i.v.	Placebo, within-subjects	20 healthy subjects, 4 females, mean age = 30.9y ± 7.8	60 min after infusion	3 T GE HDx system
Lawn et al. (2022)	LSD 75 μg i.v.	Placebo, within-subjects	15 healthy subjects, 11 females, mean age = 30.5y ± 8.0	60 min after infusion	3 T GE HDx system
Viol et al. (2017)	Ayahuasca (2.2 mL/kg containing 0.8 mg/mL of DMT and 0.21 mg/mL of harmine)	Baseline, open label	9 healthy subjects, 5 females	before and 40 min subsequent to Ayahuasca intake	1.5 T Siemens Magneton Vision
Mallaroni et al. (2024)	Ayahuasca (mean 24 mL, containing 0.14 mg/mL of DMT, 4.50 mg/mL of harmine, 0.51 mg/mL of harmaline, and 2.10 mg/mL of tetrahydroharmine)	Baseline, within-subject, fixed-order observational study	21 healthy subjects (experienced Santo Daime members), 10 females, mean age = 54.48y, SD = 10.55	1 h after intake	7 T Siemens MAGNETOM scanner
Timmermann et al. (2023)	DMT 20 mg i.v.	Placebo, single-blind, counter-balanced	20 healthy subjects, 7 female, mean age = 33.5y, SD = 7.9	Scans lasting 28 min with DMT/placebo administered at the end of 8th min and scanning being over 20 min after injection	3 T Siemens Magnetom Verio syngo MR B17
Singleton et al. (2023)	DMT 20 mg i.v.	Placebo, single-blind, counter-balanced	20 healthy subjects, 7 female, mean age = 33.5y, SD = 7.9	Scans lasting 28 min with DMT/placebo administered at the end of 8th min and scanning being over 20 min after injection	3 T Siemens Magnetom Verio syngo MR B17
Pasquini et al. (2024)	DMT 20 mg i.v.	Placebo, single-blind, counter-balanced	14 healthy subjects, 4 females, mean age = 34.1y, SD = 8.8	Scans lasting 28 min with DMT/placebo administered at the end of 8th min and scanning being over 20 min after injection	3 T Siemens Magnetom Verio syngo MR B17
Vohryzek et al. (2024)	DMT 7/14/18/20 mg i.v.	Placebo, fixed-order, single-blind	20 healthy subjects, 7 females, mean age = 33.5y, SD = 7.9	DMT/placebo bolus in 8th minute of 28-min scan	3 T Siemens Magnetom Verio syngo MR 12
Resting-state functional connectivity					
Carhart-Harris et al. (2012a)	Psilocybin 2 mg i.v.	Placebo, within-subject	15 healthy subjects, 13 males and 2 females, mean age = 32y	60 s infusion exactly 6 min after the start of the 12 min scan	3 T GE HDx system
Carhart-Harris et al. (2013)	Psilocybin 2 mg i.v.	Placebo, within-subject	15 healthy subjects, 13 males and 2 females, mean age = 32y	60 s infusion exactly 6 min after the start of the 12 min scan	3 T GE HDx system
Roseman et al. (2014)	Psilocybin 2 mg i.v. (MDMA)	Placebo, within-subject	15 healthy subjects, 13 males and 2 females, mean age = 32y	60 s infusion exactly 6 min after the start of the 12 min scan	3 T GE HDx system
Lebedev et al. (2015)	Psilocybin 2 mg i.v.	Placebo, within-subject	15 healthy subjects, 13 males and 2 females, mean age = 32y	60 s infusion exactly 6 min after the start of the 12 min scan	3 T GE HDx system
Carhart-Harris et al. (2017)	Psilocybin 10 mg and 25 mg p.o.	Baseline, open-label	16 subjects with TRD, 4 female, mean age = 42.75y, SD = 10.15	At baseline and 1 day after second dosing	3 T Siemens Tim Trio
Smigielski et al. (2019)	Psilocybin 315 μg/kg p.o.	Placebo, double-blind, randomized	38 healthy, experienced meditator subjects (20 in active group), 15 females, mean age = 51.66y, SD = 8.32	Day before and after a 5-day meditation retreat with psilocybin administered on fourth day	3 T Philips Achieva
Barrett et al. (2020a)	Psilocybin 25 mg/70 kg p.o.	Baseline, open-label	12 healthy subjects, 7 females, mean age = 32.1y, SD = 7.5	One day before, one week after, and one month after psilocybin administration	3 T Philips Achieva
Barrett et al. (2020b)	Psilocybin 10 mg/70 kg p.o.	Placebo, blinded	15 healthy subjects, 5 females, mean age = 51.3y, SD = 12.3	100 min after administration	3 T Philips Achieva
Madsen et al. (2021)	Psilocybin 0.2–0.3 mg/kg p.o.	Non-psychedelic drug, blinded	15 healthy subjects, 6 females, mean age = 34.3y, SD = 9.8	Prior and 40, 80, 130 and 300 min after psilocybin	3 T Siemens Prisma
Gaddis et al. (2022)	Psilocybin 10 mg/70 kg p.o.	Placebo, single-blind, within-subjects	18 healthy subjects, 5 females, mean age = 54.4y, SD = 13.2	100 min after administration	3 T Philips Achieva
McCulloch et al. (2022a)	Psilocybin 0.2–0.3 mg/kg p.o.	Baseline	10 healthy subjects, 4 females, mean age = 28.3y, SD = 3.4 years	15.3 ± 9.3 days before and one week and 3 months after psilocybin administration	3 T Prisma
Madsen et al. (2024)	Psilocybin 0.14 mg/kg 3 times	Baseline, open label	10 subjects with chronic cluster headache	1 day before the first psilocybin dose and 1 week after the last dose	3 T Siemens Prisma
Siegel et al. (2024)	Psilocybin 25 mg p.o. (Methylphenidate)	Baseline, methylphenidate, randomized cross-over	6 healthy subjects 18–45y	Regular MRI sessions (roughly 18 per participant) before, during (60–180 min following administration), between and after the two drug doses	3 T Siemens Prisma
Moujaes et al. (2024)	LSD 100 μg p.o., psilocybin 0.2 mg/kg p.o.	Placebo, double-blind, randomized, crossover design (both substances)	24 healthy subjects, 5 females, mean age = 25.00y; SD = 3.60 (LSD); 23 healthy subjects, 12 males, 11 females, mean age = 26.3y (psilocybin)	75 and 300 min after treatment administration (LSD); 20, 40, and 70 min after treatment administration (psilocybin)	3 T Philips Achieva
Carhart-Harris et al. (2016)	LSD 75 μg i.v.	Placebo, within-subjects	20 healthy subjects, 4 females, mean age = 30.9y ± 7.8	60 min after infusion	3 T GE HDx system
Tagliazucchi et al. (2016)	LSD 75 μg i.v.	Placebo, within-subjects	20 healthy subjects, 4 females, mean age = 30.9y ± 7.8	60 min after infusion	3 T GE HDx system
Speth et al. (2016)	LSD 75 μg i.v.	Placebo, within-subjects	20 healthy subjects, 4 females, mean age = 30.9y ± 7.8 (19 included)	60 min after infusion	3 T GE HDx system
Müller et al. (2017)	LSD 100 μg p.o.	Placebo, randomized, double-blind cross-over design	20 participants, 10 females, mean age = 32.4y, SD = 10.9	Scan starting 2.5 h after administration of placebo/LSD	3 T Magnetom Prisma, Siemens Healthcare
Müller et al. (2018)	LSD 100 μg p.o.	Placebo, randomized, double-blind, cross-over trial	20 participants, 10 females, mean age = 32.4y, SD = 10.9	Scan starting 2.5 h after administration of placebo/LSD	3 T Magnetom Prisma, Siemens Healthcare
Avram et al. (2022)	LSD 100 μg p.o. (MDMA, D-amphetamine)	Placebo, double-blind, crossover design with 4 sessions in a random and counterbalanced order	25 healthy subjects, 12 females, mean age = 28.2y, SD = 4.35	Scan starting 2.5 h after administration of placebo/LSD	3 T Magnetom Prisma, Siemens Healthcare
Dai et al. (2023)	LSD 75 μg i.v. (NO, ketamine)	Placebo, within-subjects	15 healthy subjects, 5 females, mean age = 38.4y ± 8.6	60 min after infusion	3 T GE HDx system
Pizzi et al. (2023a)	LSD 75 μg i.v.	Placebo, within-subjects	15 healthy subjects, 4 females, mean age = 30.5y ± 8.0	60 min after infusion	3 T GE HDx system
Pizzi et al. (2023b)	LSD 75 μg i.v.	Placebo, within-subjects	15 healthy subjects, 4 females, mean age = 30.5y ± 8.0	60 min after infusion	3 T GE HDx system
Shukuroglou et al. (2023)	Psilocybin 10 mg and 25 mg p.o.	Baseline, open label	16 subjects with TRD, 4 female, mean age = 42.75y, SD = 10.15	At baseline and 1 day after second dosing	3 T Siemens Tim Trio
Palhano-Fontes et al. (2015)	Ayahuasca (2.2 mL/kg containing 0.8 mg/mL of DMT and 0.21 mg/mL of harmine)	Baseline, open label	9 healthy subjects with at least 5 years of regular (twice a month) Ayahuasca use, 5 females, mean age = 29y, 24–48y	Before and 40 min after Ayahuasca intake	1.5 T Siemens, Magneton Vision
Sampedro et al. (2017)	Ayahuasca (148 ± 29 mL, containing 0.3 mg/mL DMT, 0.86 mg/mL harmine, 0.17 mg/mL tetrahydroharmine, and 0.04 mg/mL harmaline)	Baseline, open label	16 healthy subjects with prior ayahuasca experience (previously taken 62 times, SD = 99), 6 females, mean age = 38.9y, SD = 7.8	24 h prior and 24 h after the ayahuasca session	3 T Siemens Magneto TIM Trio
Pasquini et al. (2020)	Ayahuasca (1 mL/kg, containing 0.36 mg/mL of N, N-DMT, 1.86 mg/mL of harmine, 0.24 mg/mL of harmaline, and 1.20 mg/mL of tetrahydroharmine)	Placebo, blinded	50 healthy subjects (25 in active group)	1 day before and 24 h after dosing	1.5 T General Electric, HDxt
Effective connectivity					
Kraehenmann et al. (2015a)	Psilocybin 0.16 mg/kg p.o.	Placebo, randomized, double-blind, crossover design	25 healthy subjects, 9 females, mean age = 24.2y, SD = 3.42	During task	3 T Philips Achieva
Stoliker et al. (2024)	Psilocybin 0.2 mg/kg p.o.	Placebo, double-blind, randomized, crossover study	23 healthy subjects, 12 males, 11 females, mean age = 26.3y	70 min after treatment administration	3 T Philips Achieva
Kaelen et al. (2016)	LSD 75 μg i.v.	Placebo, within-subjects	20 healthy subjects, 4 females, mean age = 30.9y ± 7.8 (12 included)	60 min after infusion	3 T GE HDx system, BOLD fMRI
Preller et al. (2019)	LSD 100 μg p.o.	Placebo/ketanserin, double-blind, randomized, crossover design	25 healthy subjects, 6 females, mean age = 25.24y, SD = 3.72	75 min and 300 min after treatment administration	3 T Philips Achieva
Stoliker et al. (2023)	LSD 100 μg p.o.	Placebo, double-blind, randomized, crossover study	25 healthy subjects, 6 females, mean age = 25.24y, SD = 3.72	75 min and 300 min after treatment administration	3 T Philips Achieva
Avram et al. (2023)	LSD 100 μg p.o. (MDMA, D-amphetamine)	Placebo, double-blind, crossover design with 4 sessions in a random and counterbalanced order	25 healthy subjects, 12 females, mean age = 28.2y, SD = 4.35	Scan starting 2.5 h after administration of placebo/LSD	3 T Magnetom Prisma, Siemens Healthcare
Bedford et al. (2023)	LSD 100 μg p.o.	Placebo, randomized, double-blind, cross-over trial	20 healthy subjects, 10 females, mean age = 32.4y, SD = 10.9	Scan starting 2.5 h after administration of placebo/LSD	3 T Magnetom Prisma, Siemens Healthcare
Task-based fMRI—activation studies					
Carhart-Harris et al. (2012b)	Psilocybin 2 mg i.v.	Placebo, within-subject	15 healthy subjects, 13 males and 2 females, mean age = 32y	60 s infusion exactly 6 min after the start of the 12 min scan	3 T GE HDx system, BOLD fMRI
Kraehenmann et al. (2015b)	Psilocybin 0.16 mg/kg p.o.	Placebo, randomized, double-blind, crossover design	25 healthy subjects, 9 females, mean age = 24.2y, SD = 3.42	During task	3 T Philips Achieva
Preller et al. (2016)	Psilocybin 0.215 mg/kg p.o.	Placebo, randomized, double-blind, within-subject	21 healthy subjects, 9 females, mean age = 26.48y, SD = 4.76	During task	3 T Philips Achieva
Roseman et al. (2018)	Psilocybin 10 mg and 25 mg p.o.	Baseline, open label trial	16 subjects with TRD, 4 female, mean age = 42.75y, SD = 10.15	At baseline and 1 day after second dosing	3 T Siemens Tim Trio
Barrett et al. (2020a)	Psilocybin 25 mg/70 kg p.o.	Baseline, open-label	12 healthy subjects, 7 females, mean age = 32.1y, SD = 7.5	One day before, one week after, and one month after psilocybin administration	3 T Philips
Mertens et al. (2020)	Psilocybin 10 mg and 25 mg p.o.	Baseline, open label trial	16 subjects with TRD, 4 female, mean age = 42.75y, SD = 10.15	At baseline and 1 day after second dosing	3 T Siemens Tim Trio
Duerler et al. (2021)	Psilocybin 0.2 mg/kg p.o.	Placebo, double-blind, randomized, crossover design	15 healthy subjects, 5 females, mean age = 26.86y	85 min after psilocybin/placebo administration	3 T Philips Acheva
Pagni et al. (2024)	Psilocybin 25 mg/70 kg p.o.	Placebo-controlled	14 subjects with alcohol use disorder (6 in psilocybin group)	2–3 days before and 1–2 days after receiving the first dose of study blinded medication	3 T Siemens Skyra
Mueller et al. (2017)	LSD 100 μg p.o.	Placebo, randomized, double-blind, cross-over design	20 healthy subjects	Scan starting 2.5 h after administration of placebo/LSD	3 T Magnetom Prisma, Siemens Healthcare
Preller et al. (2017)	LSD 100 μg p.o.	Placebo/ketanserin, double-blind, randomized, crossover design	22 healthy subjects, 5 females, mean age = 25.68y, SD = 3.67	Scan 100 min after treatment with placebo or LSD	3 T Philips Achieva
Preller et al. (2018a)	LSD 100 μg p.o.	Placebo/ketanserin, double-blind, randomized, crossover design	24 healthy subjects, 6 females, mean age = 25.42y, SD = 3.6	Task conducted 310 min after treatment administration	3 T Philips Achieva
Schmidt et al. (2018)	LSD 100 μg p.o.	Placebo, randomized, double-blind, cross-over design	18 healthy subjects, 9 females, mean age = 31y, SD = 9	Task conducted 200 min after drug administration	3 T Siemens Magnetom Verio
Duerler et al. (2020)	LSD 100 μg p.o.	Placebo/ketanserin, double-blind, randomized, crossover design	24 healthy subjects, 6 females, mean age = 25.25y, SD = 3.72	Task conducted 330 min after treatment administration	3 T Philips Achieva
de Araujo et al. (2011)	Ayahuasca (2.2 mL/kg containing 0.8 mg/mL of DMT and 0.21 mg/mL of harmine)	Baseline, open label	9 healthy subjects, frequent Ayahuasca users, 5 females, mean age = 29y (24 to 48y)	Before and 40 min after ayahuasca intake	1.5 T Siemens Magneton Vision
Arruda Sanchez et al. (2024)	Ayahuasca (30 mL/70 kg, corresponding to 0.333 mg/kg DMT, 0.473 mg/kg Harmine, 0.023 mg/kg Harmaline, and 0.470 mg/kg THH)	Baseline, open label	19 healthy subjects, experienced Ayahuasca users, all males, mean age = 31.5y, SD = 10.7	Before and 50 min after ayahuasca ingestion	1.5 T Siemens Magnetom Avanto

While the studies within the field remain very heterogenous, owing in part to the breadth of problems possible to study *through* psychedelics, we have indeed been able to identify several main themes which repeat in variations across studies and sometimes different substances. Some of these have already been so widely replicated that they have their firm place in the current discourse (e.g., decrease in modularity, increase in global connectivity), some correspond to the respective models of psychedelic action described above, while others could be described as more outlying.

### 3.1 Global measures

We begin with description of the effects psychedelics exert on the brain as a whole. Among these, the clearest finding is a general increase in global functional connectivity, found across multiple psychedelic substances (Timmermann et al., 2023; Tagliazucchi et al., 2016); in some cases, the findings become more nuanced, observing a simultaneous increase in sensory global connectivity and decrease in associative global connectivity (Preller et al., 2018b, 2020). Global brain hyperconnection was also linked to heightened subjective effects (Mortaheb et al., 2024).

Another widely discussed finding is an increase in entropy, with one study even finding a correlation with an increase in openness after 2 weeks (Lebedev et al., 2016). Entropy is a quantity originally derived from thermodynamics, broadly defined as a measure of uncertainty of a system, or the degree of its disorder—in neurobiological sense, this degree of disorder has been posited to reflect the measure of subjective uncertainty and ultimately level of consciousness (Carhart-Harris et al., 2014). It can also be viewed as a measure of complexity (Mikoláš et al., 2012). However, entropy remains an elusive metric, with a number of different techniques employed to determine it (see McCulloch et al., 2024) for their discussion and evaluation. These include Shannon entropy (Viol et al., 2017), sample entropy (Lebedev et al., 2016), entropy of possible states (Tagliazucchi et al., 2014) or examining complexity more generally through brain activity fractal dimension analysis (Varley et al., 2020). In addition, entropy is explored in EEG and MEG studies (Schartner et al., 2017)—these however are beyond the scope of this paper.

Apart from these findings, there is a number of other experimental approaches to examining global brain function, such as studies on brain dynamics, which investigate temporal changes within the fMRI time series. Consistent with the findings of increased entropy, a series of studies with psilocybin, LSD (Singleton et al., 2022) and DMT (Singleton et al., 2023) utilizing network control theory has found a reduction of energy needed for transitioning from one brain state (here defined as commonly recurring patterns of co-activation) to another, thus allowing for more facile switching between them. Leading Eigenvector Dynamics Analysis (LEiDA), a method examining time-varying functional connectivity patterns, has revealed a suppression of a pattern corresponding to the fronto-parietal network and at the same time a state of increased global coherence, suggestive of weakening of the normally present network structure and instead transition into globally highly integrated state (Lord et al., 2019); a more recent study then replicated these findings and found their correlation with psilocin plasma levels and subjective drug effects (Olsen et al., 2022). General de-differentiation (we define de-differentiation here as a reduction in normally present difference in function of distinct brain regions or networks) of brain function was shown by a finding of a compression of a gradient of cortical hierarchy across different psychedelic substances (Girn et al., 2022; Timmermann et al., 2023). Multiple studies have also explored connectome harmonics in different substances, decomposing brain activity into harmonic states to explore the functional connectome, and finding a move toward higher frequencies representing a divergence from structural connectome—those findings can be interpreted as a move toward criticality (Atasoy et al., 2017; Atasoy et al., 2018). The most recent study in this area conducted with DMT also found correlation of these effects with subjective intensity of the experience (Vohryzek et al., 2024).

In sum, these heterogenous studies show functional de-differentiation of the brain with increased entropy and more fluid dynamics. These results can be interpreted as consistent with the REBUS hypothesis, which posits a highly malleable state of the brain during the psychedelic experience. Further work is now needed in reducing this heterogeneity (e.g., through replication) to bring about a more coherent and rigorous basis for the REBUS model (McCulloch et al., 2024).

### 3.2 Resting-state functional connectivity

As described above, the brain is ordered into a number of resting-state networks under normal circumstances. Among major of these, the central executive network (CEN) is associated with focused attention, in contrast to the default mode network being associated with introspection; the activity of these two networks is thus anticorrelated, with the salience network posited to switch between them (Nekovarova et al., 2014). Possibly the most consistent finding within the fMRI of the psychedelic state is a general dissolving of this brain modularity—a decrease in connectivity within the resting-state networks, and increase in connectivity between them. This has been widely replicated across different studies and different substances (Müller et al., 2018; Madsen et al., 2021; Timmermann et al., 2023; Roseman et al., 2014). In line with these findings, network integrity has been observed to negatively correlate with psilocin plasma levels and its subjective effects (Madsen et al., 2021). Significantly, in a set of patients with treatment-resistant depression where psilocybin was effective in treating their symptoms, this alleviation correlated with the decrease in network modularity after dosing (Daws et al., 2022).

This modularity decrease seems to be the most pronounced in the DMN: its disintegration correlates with subjective measures of ego-dissolution and with an improvement in psychosocial functioning (Smigielski et al., 2019). In a study with ayahuasca, the decrease of connectivity within the DMN lingered even 24 h after dosing (Pasquini et al., 2020). In line with the general breakdown of standard DMN functioning, there is a decrease of its anticorrelation with the task-positive networks, normally a defining feature of the DMN (Carhart-Harris et al., 2013); a reduction of functional connectivity between its components was one of the very first findings within the psilocybin fMRI studies (Carhart-Harris et al., 2012a). Underscoring the importance of the DMN, a recent longitudinal study found a decrease of its connectivity with anterior hippocampus, another key structure implicated in the psychedelic state discussed below, lasting for 3 weeks after dosing (Siegel et al., 2024).

A second major finding that has been widely replicated and discussed is an increase in connectivity between the thalamus and the cortex (Müller et al., 2018; Tagliazucchi et al., 2016; Preller et al., 2018b) (see also the general CSTC model Vollenweider and Geyer, 2001; Avram et al., 2021), with individual studies finding associations with subjective sensory effects (Avram et al., 2022; Müller et al., 2017). However, this connectivity pattern seems to be more complicated with a closer look: more detailed analyses of thalamic connectivity bring conflicting and more nuanced results. While one study with LSD found increased connectivity of the pulvinar and ventral nuclei of thalamus with the cortex (Pizzi et al., 2023b), a 7 tesla MRI study pinpointing the effect of psilocybin on the thalamus to the pulvinar and the mediodorsal nucleus surprisingly found a *decrease* in connectivity of these structures with cortical areas—consistent with these nuclei maximally expressing the 5HT2A receptor within the thalamus (Gaddis et al., 2022).

Among other specific brain structures, there are numerous changes in the medial temporal lobe structures, with a reduction of connectivity of the parahippocampal cortex correlating with ego dissolution (Carhart-Harris et al., 2016), an already mentioned longitudinal decrease in connectivity of the hippocampi with the DMN (Siegel et al., 2024) and an increase of signal variability and entropy within them (Tagliazucchi et al., 2014); all of these studies being done with psilocybin, while an infusion of DMT produced a deactivation of the hippocampi, which correlated with subjective meaningfulness of the experience (Pasquini et al., 2024). Apart from the hippocampus, the claustrum has been implicated in psychedelic drug action, with alterations of its connectivity to the DMN, frontoparietal and auditory networks (Barrett et al., 2020b), forming the basis of the CCC model (Doss et al., 2022); these findings have recently been corroborated by a nonhuman primate study which found psilocybin-induced increase in connectivity of the claustrum with the anterior cingulate cortex, the precuneus and areas of the prefrontal cortex (Bagdasarian et al., 2024).

Taken together, studies on functional connectivity broadly show dissolving of resting-state networks most prominent in the DMN, increase of thalamocortical connectivity with a need for further clarification, decreases in connectivity of medial temporal lobe structures and alterations of connectivity of the claustrum. The CCC model, while intriguing, is currently still based on only one human study, making replication indispensable to prove its validity—the increasing use of higher-resolution MRI scanners (see Table 2) has the potential to make studying this difficult-to-measure structure more accessible.

### 3.3 Task-based fMRI—activation studies

A large number of activation studies have been conducted as well, with intriguing but highly heterogenous paradigms and results, making any attempt at generalization difficult. A closely examined brain region is the amygdala, a key structure of emotional processing that in depression has been demonstrated to be hyperactive in response to negative stimuli and hypoactive in response to positive stimuli (Stuhrmann et al., 2011), with this finding being attenuated by SSRI treatment (Ma, 2015). With psychedelics, multiple studies with psilocybin, LSD and ayahuasca observe a decrease of its activity in response to negative stimuli (Barrett et al., 2020a; Kraehenmann et al., 2015b; Arruda Sanchez et al., 2024); in one study, this correlated with the intensity of the subjective effects (Mueller et al., 2017). A study of patients with treatment-resistant depression found an increase of amygdala reactivity to both fearful and happy faces (in contrast to neutral faces), with this phenomenon predictive of alleviation of depression in the first week after dosing (Roseman et al., 2018). Thus, these findings seem to validate the potential of psychedelics to treat depression, with an interesting difference of the finding of *increased* reactivity to happy *and* fearful faces in the population of patients with depression. Additionally, changes to effective connectivity of the amygdala have been identified by two studies discussed in the following section.

### 3.4 Effective connectivity

**Figure 2:**
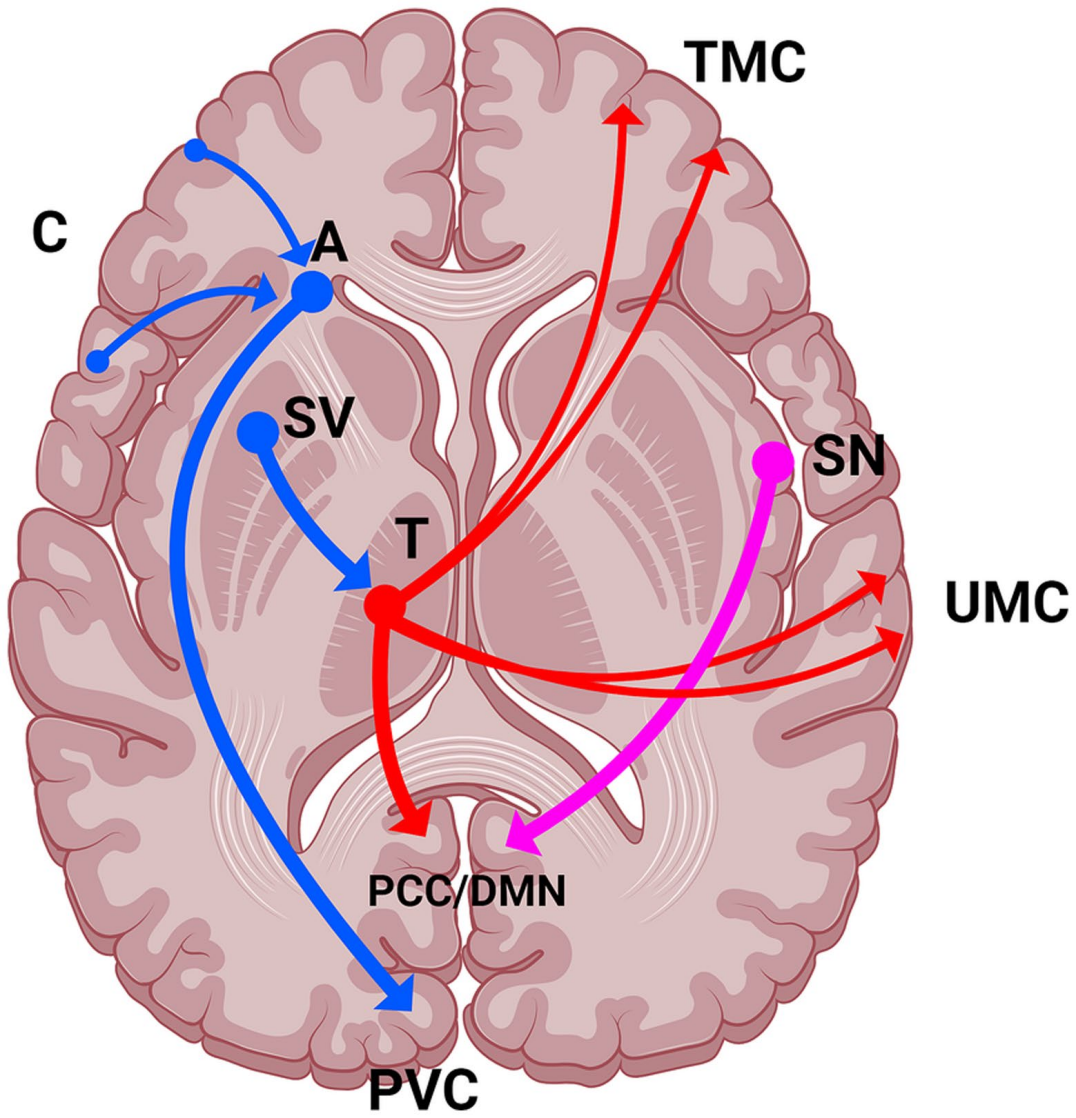
Schematic summary of major findings of effective connectivity changes. Blue neurons represent decreases in EC, red neurons represent increases in EC, pink neurons represent change from inhibitory EC to excitatory. The direction of effective connectivity is represented by the arrow. Study with music not shown. A, amygdala; C, cortex; DMN, default mode network; PCC, posterior cingular cortex; PVC, primary visual cortex; SN, salience network; SV, striatum ventrale; T, thalamus; TMC, transmodal cortex; UMC, unimodal cortex.

Findings of functional connectivity changes described above can be further expanded by exploring effective connectivity (EC). Building on the CSTC model, LSD has been found to increase EC from the thalamus to posterior cingulate cortex (a part of the DMN) while also decreasing EC from striatum ventrale to the thalamus (Preller et al., 2019); it was also shown to increase EC from the thalamus to both unimodal and transmodal cortex, contrasted to MDMA and amphetamine which increased EC only to the unimodal cortex, highlighting a possible breach in cortical hierarchy (see the section on global measures) as a feature unique to psychedelics (Avram et al., 2023). Expanding on the picture of complex interplay between the main resting-state networks with the central role of the DMN, LSD was shown to flip the valence of connectivity (inhibitory to excitatory) from the salience network to the DMN, and decrease the inhibitory connectivity from the DMN to the dorsal attention network (Stoliker et al., 2023). Lastly, the knowledge on changes to amygdala was expanded by two studies: (a) psilocybin was shown to weaken modulatory effect of visual threat on the connection from the amygdala to primary visual cortex (Kraehenmann et al., 2015a) and (b) psilocybin decreased EC from cortical regions to the amygdala, with the notable exception of the DMN (as well as to alter EC within the DMN, CEN, and SN), suggesting changes to hierarchical organization of brain function (Stoliker et al., 2024). See Figure 2 for schematic summary of effective connectivity changes.

Together with functional connectivity investigations, effective connectivity studies put the CSTC model on a solid footing, showing the actual flow of information in the expected direction and highlighting its breadth compared to non-psychedelic drugs. Regarding the model in general, clarifications are needed as to precisely which cortical regions are affected; an attempt at replicating the Gaddis et al. (2022) study is warranted, as its findings challenge the model in its current form.

### 3.5 fMRI correlates of ego dissolution

Lastly, outsize importance of ego dissolution, a specific phenomenon within the psychedelic experience characterized by a breakdown of the sense of self (Stoliker et al., 2022), is becoming clear through fMRI studies. In itself, ego dissolution is not an fMRI concept, but a subjective phenomenon—in psychedelic research these are generally ascertained by psychometric scales, such as the Five Dimensional Altered States of Consciousness (5D-ASC). This scale groups the subjective elements of the psychedelic experience into 3 main themes (Visionary Restructuralization, Oceanic Boundlessness and Anxious-Ego Dissolution) with 11 subthemes among these. We choose to include ego dissolution as a section here as there are multiple fMRI findings associated specifically with it, and it has connections beyond them, with its extent shown to predict positive changes in psycho-social functioning 4 months after dosing of psilocybin (Smigielski et al., 2019). In relation to fMRI, its occurrence is specifically associated with a decrease in connectivity between the parahippocampal cortex and retrosplenial cortex (Carhart-Harris et al., 2016), decrease in connectivity between the medial temporal lobe and the cortex, disintegration of the salience network and a decrease in interhemispheric communication (Lebedev et al., 2015). The changes observed by Stoliker et al. (2023) described in the section on effective connectivity also correlated with measures of ego dissolution. In addition, an increase in global connectivity (Tagliazucchi et al., 2016) and disintegration of the DMN (Smigielski et al., 2019) have been observed specifically during ego dissolution, both in line with more general findings during the psychedelic experience, suggesting their deepening during this phenomenon. However, as stated above, fMRI correlates are just one of many aspects of ego dissolution—for a comprehensive review of its mechanisms see Stoliker et al. (2022).

## 4 Discussion

Taken together, a look at the entirety of the studies reviewed offers a rich and diverse landscape of findings, where lines of consistency are starting to take shape. These lines are present both “horizontally” in the form of converging results we have highlighted here, but also “vertically,” in the form of associations with findings outside of the fMRI sphere: namely with the subjectively reported effects, drug plasma levels, and also different neuroimaging modalities [e.g., employment of simultaneously recorded EEG-fMRI (Timmermann et al., 2023)]. Overall, this consistency is putting the fMRI findings on firmer footing. Another instance of this could be seen in tracking of longitudinal changes, with well-known studies that show persisting effects through subjective measures (Griffiths et al., 2018) and through disease symptom remission (Carhart-Harris et al., 2021) recently being shored up by findings of fMRI changes persisting as well (Siegel et al., 2024; Pasquini et al., 2020; McCulloch et al., 2022a; Barrett et al., 2020a). Given the time course of mental disorders potentially treatable by psychedelics, possibility of their relapse and discussions of deeper personality change through psychedelic treatment, further ascertaining the specifics of these long-term effects will be a crucial direction moving forward.

Further research into subdivision of the psychedelic state itself also appears crucial—this is exemplified by studies on ego dissolution, which generally show specific changes unique to this phenomenon and not present in the psychedelic state as a whole, and which have been shown to translate into long-term effects (Smigielski et al., 2019), demonstrating their significance. An example of advancing this line of thinking can be seen in recent studies on DMT (Timmermann et al., 2023), which take advantage of its short half-life allowing to capture the whole or most of the psychedelic state within the session.

With progress in the MR technology itself, findings suggestive a new level of complexity are emerging and showing the necessity of further clarification to established findings, such as the discovery of decreased thalamocortical connectivity when looking at individual thalamic nuclei by Gaddis et al. (2022). Further, as new approaches are also being developed in the area of global measures, the overarching picture of general de-differentiation of brain function keeps emerging through a wide variety of different techniques—this would seem to be broadly consistent with the REBUS hypothesis, and speculatively with the potential of psychedelics being able to act transdiagnostically (Kočárová et al., 2021; Carhart-Harris et al., 2023). Further work on integrating the models of action together is warranted.

Lastly, a meaningful route of filtering out noise and exploring neurobiological undercurrents appears to be one of drawing contrasts between psychedelics and phenomenologically similar yet neurobiologically different states: (a) those elicited by psychoactive substances other than classical psychedelics, such as the finding that MDMA (and amphetamine) increase effective connectivity to unimodal cortex contrasted with LSD increasing EC also to transmodal cortex (Avram et al., 2023), of methylphenidate (which was used in the study as an active control) showing changes corresponding to general arousal contrasted with more specific changes of psilocybin (Siegel et al., 2024), or of ketamine (and NO) producing similar changes (decrease in within-network connectivity and increase in between-network connectivity) to LSD (Dai et al., 2023); and (b) altered states of consciousness such as meditation, hypnosis or schizophrenia. Specifically, the parallels with schizophrenia form an avenue of research as old as the psychedelic substances themselves (Nichols, 2016), which is promising inways into two states that both warrant more complete understanding, as well as into more fundamental concepts of neuroscience and psychology such as the self, of which there are clear alterations in both conditions (Kozáková et al., 2020; Stoliker et al., 2022). There has been a constant stream of studies drawing these parallels with schizophrenia, many of which are consistent with the themes explored in this review: altered thalamocortical connectivity (Avram et al., 2021), disruption to the DMN-task positive network anticorrelation (Carhart-Harris et al., 2013) and principal cortical gradient compression (Girn et al., 2022; Timmermann et al., 2023). Another compelling parallel could be represented by the deep disruptions within the triad of DMN, central executive and salience network, which have also been described in schizophrenia (Nekovarova et al., 2014). For further discussions on this topic (see also Leptourgos et al., 2020; Sapienza et al., 2023). As for meditation, previous studies have intriguingly shown changes in similar areas compared to psychedelics, such as more robust DMN activation compared to rest (Xu et al., 2014) and subacute increase in connectivity of the DMN with the dorsal attention network and visual cortex (Zhang et al., 2021); see Boccia et al. (2015) for a meta-analysis. For a direct comparison of fMRI correlates of the psychedelic state, hypnosis and meditation see Moujaes et al. (2024).

From a methodological standpoint and as highlighted by McCulloch et al. (2022b), there is a great need for more independent replication of the available findings, as the studies available come from a limited number of relatively small datasets. To this end, focus on data standardization will be crucial moving forward, as currently there is a great degree of heterogeneity in multiple aspects. Scan timepoints vary, and while each of them may be justified (or simply necessary, e.g., with the need to fit multiple tasks into one administration session), the resulting data originate from different sections of the psychedelic experience—this can be a complication for instance in studying ego dissolution, as it is a phenomenon that occurs only during a fraction of the whole experience. Further complicating matters, drug administration methods vary, with both per os and i.v. being utilized, resulting in different plasma level dynamics for same substances and thus making it difficult to pinpoint equivalent scan timepoints. Another potential source of heterogeneity lies in fMRI data processing, for instance in using in-house pre-processing pipelines, which by definition vary between institutions, and can for example influence whether a given result reaches the level of statistical significance or not. Finally, subject characteristics can be a source of heterogeneity—in a large part of the ayahuasca studies, participants were experienced users, while long term use of ayahuasca has been shown to elicit structural changes (specifically, thinning of the posterior cingulate cortex) in the brain (Bouso et al., 2015), potentially leading to different fMRI findings compared to first-time users; other example would be potential differences between clinical populations and healthy volunteers. In sum, this heterogeneity will need to be taken into account in studies aiming to replicate these results.

## 5 Limitations

There are limitations to this study to be mentioned here. While our aim was to offer a conceptual overview of the field, this led to a large number of existing studies not being mentioned in the narrative text for the sake of clarity. Furthermore, with the decision to mostly treat the serotonergic psychedelics as a whole also necessary to formulate a somewhat digestible picture of the field, it is naturally an oversimplification, with significant differences among the substances in themselves. Perhaps the most obvious of these would be different pharmacokinetics, for instance half-life (approximately 4 h for LSD, compared to only 5–15 min for DMT), but also speed of onset and offset, adding to the heterogeneity discussed above (Holze et al., 2024). Differences also lie in subjective effects, in the simplest form in perceived intensity of the experience, but also in more nuanced phenomenological aspects—for instance in the form of perceived entity encounters, which is a phenomenon characteristic for DMT. There are also nuances in receptor profiles of the substances. Generally, these differences could be explored by further research combining fMRI with other modalities: namely, studies with PET (both FDG and specific receptor ligands) and EEG. Also refer to Frautschi et al. (2024) for a discussion of this heterogeneity.

As for methodological limitations, only one database (PubMed) was used to search for articles—however, given that it is a database most relevant in the research field (neuroscience with overlap into psychiatry) and we did not aim to review entries such as conference abstracts or case reports, we do not consider this a major limitation. Risk of bias assessment and review protocol pre-registration were not performed for this scoping review.

## 6 Conclusion

In conclusion, we show that numerous changes to brain function induced by serotonergic psychedelics have been observed using fMRI. The converging findings with the strongest evidence are: a general increase in connectivity throughout the brain, increase in entropy, dissolution of resting-state brain networks which is most pronounced in the DMN, increased thalamocortical connectivity, decreased amygdala reactivity to negative stimuli, changes to medial temporal lobe structures, and the significance of ego dissolution. In addition, a diverse array of other individual results show possible directions of further research. Independent replication of these findings and their further validation through associations with drug plasma levels, subjective and longitudinal effects, as well as with findings from other imaging modalities, will be necessary moving forward.

## Data Availability

The original contributions presented in the study are included in the article/supplementary material, further inquiries can be directed to the corresponding author.
